# Mycoplasma detection and elimination are necessary for the application of stem cell from human dental apical papilla to tissue engineering and regenerative medicine

**DOI:** 10.1186/s40824-015-0028-0

**Published:** 2015-03-19

**Authors:** Byung-Chul Kim, So Yeon Kim, Yong-Dae Kwon, Sung Chul Choe, Dong-Wook Han, Yu-Shik Hwang

**Affiliations:** Department of Maxillofacial Biomedical Engineering and Institute of Oral Biology, School of Dentistry, Kyung Hee University, 1 Hoegi-dong, Dongdaemun-gu, Seoul, 130-701 Republic of Korea; Department of Oral Maxillofacial Surgery, School of Dentistry, Kyung Hee University, Seoul, 130-701 Republic of Korea; Department of Pediatric Dentistry, School of Dentistry, Kyung Hee University, Seoul, 130-701 Republic of Korea; Department of Cogno-Mechatronics Engineering, Pusan National University, Busan, 609-735 Korea

**Keywords:** Dental papilla, Stem cell, Mycoplasma, Proliferation, Differentiation, Osteogenic, Neural

## Abstract

**Background:**

Recently, postnatal stem cells from dental papilla with neural crest origin have been considered as one of potent stem cell sources in regenerative medicine regarding their multi-differentiation capacity and relatively easy access. However, almost human oral tissues have been reported to be infected by mycoplasma which gives rise to oral cavity in teeth, and mycoplasma contamination of ex-vivo cultured stem cells from such dental tissues and its effect on stem cell culture has received little attention.

**Results:**

In this study, mycoplama contamination was evaluated with stem cells from apical papilla which were isolated from human third molar and premolars from various aged patients undergoing orthodontic therapy. The ex-vivo expanded stem cells from apical papilla were found to express stem cell markers such as Stro-1, CD44, nestin and CD133, but mycoplama contamination was detected in almost all cell cultures of the tested 20 samples, which was confirmed by mycoplasma-specific gene expression and fluorescence staining. Such contaminated mycoplasma could be successfully eliminated using elimination kit, and proliferation test showed decreased proliferation activity in mycoplasma-contaminated cells. After elimination of contaminated mycoplasma, stem cells from apical papilla showed osteogenic and neural lineage differentiation under certain culture conditions.

**Conclusion:**

Our study proposes that the evaluation of mycoplasma contamination and elimination process might be required in the use of stem cells from apical papilla for their potent applications to tissue engineering and regenerative medicine.

## Background

Tooth morphogenesis is known to be initiated from the cellular interactions between ectoderm-derived oral epithelial cells and neural crest-derived mesenchymes [1,2], and stem cell researches to generate osteoblasts and neurons have been done extensively using stem cells from dental tissues with such neural crest-derived ectomesenchyme origin [3-5]. To dates, various postnatal stem cells have been successfully isolated from the collected dental tissues such as dental pulp, periodontal ligament and dental papilla via non-invasive procurement process [3,6,7]. During tooth development, dentin and pulp tissue are known to be developed from dental papilla, which dental papilla is evident to have a pool of stem cells with high regeneration ability [8,9], and the majority of stem cell within dental papilla has been reported to be of neural crest-derived ectomesenchyme origin [10]. From these developmental origin, relatively easy accessibility of stem cell source from discarded tooth and possible autologous implantation via cryopreservation in dental stem cell bank, dental papilla has been suggested as potent stem cell source in regenerative medicine. For instance, in the field of tissue engineering and regenerative medicine, bioengineered tooth with the correct tooth structure was developed using stem cells from molar tooth germ tissue [11], *in vivo* bone formation could be generated via transplantation of tissue engineered bone tissue formed by cryopreserved dental stem cells [12,13], and the generation of functional neurons were reported to be from dental stem cell under neural inductive cues [14].

**Figure 1:**
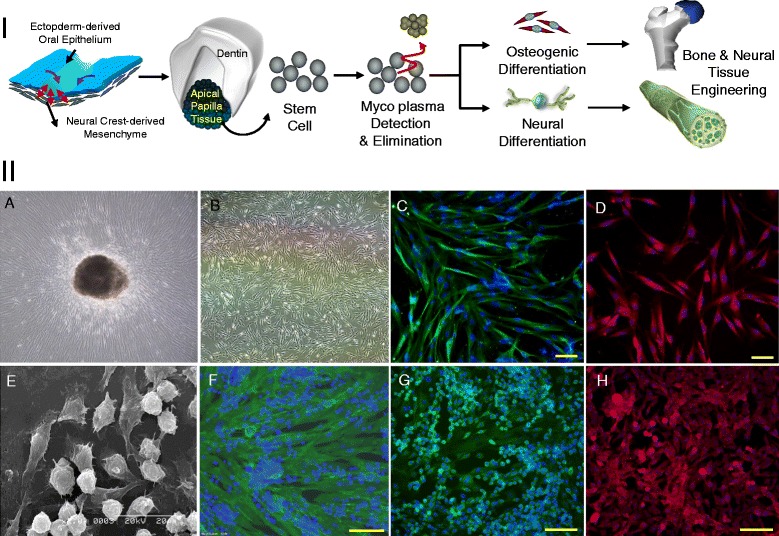
**Schematic illustration of the applications of hSCAPs to bone and neural tissue engineering and characterization of primary cultured hSCAPs.** **I**. Schematic illustration of bone and neural differentiation of mycoplasma eliminated hSCAPs for bone and neural tissue engineering. **II**. Morphology and immunocytochemical images of the primary cultured stem cells from apical papilla (hSCAPs). **A**. Cell outgrowth from apical papilla tissue fragment. **B**. Expanded hSCAPs. **C**. Stro-1(green) and nuclear staining (DAPI; blue). **D**. CD44 (red) and nuclear staining (DAPI; blue). **E**. SEM image of the expanded hSCAPs in the presence of NGF, FGF2 and LIF. **F**. CD44 (green) and nuclear staining (DAPI; blue) of the expanded hSCAPs in the presence of NGF, FGF2 and LIF. **G**. Nestin (green) and nuclear staining (DAPI; blue) of the expanded hSCAPs in the presence of NGF, FGF2 and LIF. **H**. CD133 (red) and nuclear staining (DAPI; blue) the expanded hSCAPs in the presence of NGF, FGF2 and LIF.

However, in spite of high applicability of stem cells from dental tissues in tissue engineering and regenerative medicine, mycoplasma contamination of the primary cultured stem cells from dental tissues where is easily infected by oral bacteria has been overlooked, and the evaluation and removal of infected mycoplasma in dental stem cells need to be considered, regarding biological safety in dental stem cells applications to regenerative medicine. It was reported that almost human oral tissue was frequently found to be infected small microorganism such as mycoplasma [15], and many kinds of bacteria give rise to oral cavity in teeth, and mycoplasma, the smallest and simplest self-replicating organisms, is known as one of major bacteria found in oral cavity [16]. Postnatal stem cells from infected dental tissues are evident to be infected by mycoplasma, and such mycoplama infection in dental tissues might influence dental tissue-derived stem cell’s behavior including cell proliferation. It is well known that mycoplasma infection influenced cell proliferation, chromosomal aberration in cells and induced immunological reactions [17,18]. Hence, in this study, postnatal stem cells were primarily isolated and cultured from apical papilla of the third molar and premolar teeth from various aged patients undergoing orthodontic therapy, and mycoplasma contamination was evaluated for each isolated stem cells from apical papilla (hSCAPs) of human teeth. The cell proliferation capacity was also tested with mycoplasma-infected and -eliminated hSCAPs, and then 2D and 3D osteogenic and neural differentiation capacities of mycoplasma-eliminated hSCAPs were evaluated for the potent application to bone and neural tissue engineering (Figure 1 I).

## Method

### Primary culture of Stem Cells from Apical Papilla (hSCAPs) of the human premolar and third molar teeth

Briefly, human dental papilla tissue was procured from discarded 6 ~ 24 aged donor’s premolar and third molar teeth with informed consent of patients undergoing routine extractions at the Dental Clinic of School of Dentistry in Kyung Hee University, under approved guidelines set by the Kyung Hee University and School of Dentisty Human Subjects Research Committees (IRB# KHUSD 0908–01). The extracted teeth were directly stored in alpha-MEM (Lonza) containing 1% penicillin/streptomycin (P/S, Lonza), and the teeth were used to procure papilla tissue within 2 hours after teeth extraction. Dental apical papilla tissues were extracted from the premolar and third molar teeth, and were minced with a scalpel. The fragmented papilla tissues were washed with phosphate buffered saline (PBS: Gibco) three times, and the minced tissues were allowed to attach on T25 tissue culture flasks in basic culture medium consisting of alpha-MEM, 10% fetal bovine serum (FBS, lonza), and 1% penicillin/streptomycin. Cultures were fed every 2 days and passaged by treatment with 0.25% trypsin/EDTA. Cell Cultures were grown at 37°C in a humidified atmosphere containing 5% CO_2_.

### Morphological and immunocytochemical characterization of hSCAPs

1 × 10^4^ cell/cm^2^ of hSCAPs were seeded on 24 well plate (Corning) and cultured for 2 days. The attached cells were fixed with 3.7% formaldehyde (Sigma) for 20 mins at room temperature, and fixed cells were washed with PBS. The cells were permeabilized with 0.2% Triton X-100 (Sigma) treatment for 20 mins, and, after brief washing with PBS, were treated with 4% bovine serum albumin (BSA: Sigma) to block non-specific binding of antibodies at 4°C for overnight. After blocking, the cells were incubated with primary antibodies such as 1:200-diluted mouse-anti-nestin (Abcam) antibody, 1:200-diluted mouse-anti-Stro-1 (Abcam) antibody, and 1:200-diluted mouse-anti-CD44 (Abcam), and 1:200-diluted mouse-anti-CD133 (Abcam) at 4°C for overnight. After reaction with primary antibodies, the cells were washed gently with PBS three times, and then incubated with secondary antibodies such as 1:2000-diluted alexa 488 (goat anti-mouse IgG, Invitrogen), 1:2000-diluted alexa 596 (goat anti-mouse IgG, Invitrogen) for 1 hr at room temperature under dark condition. Finally, after brief washing with PBS three times, the cells were counter-stained with DAPI, and mounted. The stained cells were observed under an inverted fluorescence microscope (Olympus, IX-72).

### Detection of mycoplasma contamination

Mycoplasma contamination of primary cultured hSCAPs was evaluated by mycoplasma specific staining method and polymerase chain reaction (PCR) method.

For mycoplasma staining, cell suspension was prepared by trypsin-EDTA treatment for 5 minutes and subsequent neutralization with culture media. Mycoplama staining was processed with prepared hSCAPs suspension and Mycoplasma Detection Kit according to manufacturer’s instruction. Briefly explaining, 95 μl of cell suspension (1 × 10^5^ cell/ml) was mixed with 5 μl of MycoFluor™ Mycoplasma Detection Kit (Invitrogen) in micro-centrifuge tube, and the mixed cell suspension was incubated for 30 mins at 37°C in a 5% CO_2_ incubator. After incubation, the stained sample was observed under an inverted fluorescence microscope (Olympus, IX-72).

To evaluate mycoplasma contamination in gene level, PCR was processed with BioMycoX Mycoplasma PCR Detection Kit (Cells-safe) according to manufacturer’s instruction. Briefly, after 24 hrs of culture, 1 ml of culture media was collected, and centrifuged at 3000 rpm for 5 mins. After centrifuge, supernatant was transferred to microcentrifuge tube, and was centrifuged at 13,000 rpm for 10 mins. After centrifuge, mycoplasma pellet was collected, and suspended with 100 μl of dH_2_O, and then boiled at 98°C for 10 mins. 50 μl of supernatant was transferred into new PCR tubes. For PCR reaction, supernatant was mixed with 5 μl PCR template, 2 μl primer mix (Cells-safe), 2 μl dH_2_O and 10 μl 2X PCR premix (Cells-safe). PCR reactions were performed as following conditions: pre-denaturation process at 95°C for 5 mins with 1 cycle, denaturation process at 94°C for 30 secs with 35 cycles, annealing process at 55°C for 30 secs with 35 cycles, and extension process at 72°C for 30 secs with 35 cycles. Finally, all PCR products were loaded on 0.7% argarose gel containing ethidium bromide at concentration of 0.5 μg/ml.

### Mycoplasma elimination

Mycoplasma elimination of contaminated hSCAPs was processed using BioMycoX® Mycoplasma Elimination Kit (Cells-safe). After detection of mycoplasma contamination, 5 × 10^6^ of the mycoplasma-infected hSCAPs was seeded on a T75 flask and cultured in culture media at 37°C in a 5% CO_2_ incubator. When the confluence of cells reached at 70 ~ 80%, the cells were trypsinized, neutralized with culture media, and then centrifuged at 1500 rpm for 5 mins. After centrifuged, cell pellet was collected, and then suspend with culture media containing 5% FBS. 2.5 × 10^6^ cells/ml was mixed with 200 μl of BioMycoX® Reagent 1, and total volume was adjusted to 10 ml with culture media. The cell suspension was transferred to a T75 flask, and incubated for 3 days without media change at 37°C in a 5% CO_2_ incubator. After 3 days of incubation, attached cells were trypsinized, and then centrifuged at 1500 rpm for 5 mins. After centrifuge, cell pellet was collected and suspended with culture media containing 10% FBS. Cell suspension was mixed with 200 μl of BioMycoX® Reagent 2, and total volume was adjusted to 10 ml with culture media. The cells were incubated for 2 days without media change at 37°C in a 5% CO_2_ incubator, which treatment with BioMycoX® Reagent 2 was repeated two times.

### Proliferation assay of mycoplasma-infected and -eliminated hSCAPs

Mycoplasma-infected hSCAPs and mycoplasma-eliminated hSCAPs were seeded at cell density of 1 × 10^4^ cells on 96 well plate and incubated for 24 hrs at 37°C in a 5% CO_2_ incubator. The BrdU incorporation based cell proliferation assay was processed with BrdU assay kit (Merck) according to manufacturer’s instruction. Briefly, after 24 hrs of incubation, 1:2000 diluted BrdU working solution was added into hSCAPs culture media, and the cells were incubated for 3 hrs. The BrdU incorporated cells were treated with 200 μl of fixative/denaturing solution for 30 mins at room temperature. After reaction, fixative/denaturing solution was aspirated, and the cells were incubated with 1:100 diluted mouse anti-BrdU in dilution buffer solution for 1 hr at room temperature. After washing with washing buffer three times, the cells were reacted with 100 μl goat anti-mouse IgG HRO conjugate for 30 mins at room temperature, and then washed with washing buffer three times. Finally, the cells were reacted with 100 μl of substrate solution in the dark at room temperature for 15 mins, and subsequently 100 μl of stop solution was added to each well. The content of incoporated BrdU in cellular DNA was measured using a spectrophotometric plate reader at dual wavelengths of 450–540 nm (or 450–595).

### 2D and 3D osteogenic differentiation of mycoplasma-eliminated hSCAPs

Prior to osteogenic differentiation, primary cultured hSCAPs were expanded in α-MEM supplemented with 15% FBS, and 1% penicillin/streptomycin. For osteogenic differentiation, hSCAPs was seeded on culture plated at cell density of 1 × 10^5^/cm^2^ and cultured in osteogenic medium; α-MEM supplemented with 15% FBS, and 1% penicillin/streptomycin containing 50 μg/ml ascorbic acid (Sigma) in PBS, 1 μM dexamethasone (Sigma) in PBS and 10 mM β-glycerophosphate (Sigma) in PBS. The cultures were maintained at 37°C in a 5% CO_2_ humidified incubator and fed every 2 days up to 20 days. After 20 days of osteogenic culture, the differentiated cells were fixed with 3.7% formaldehyde for 1 min or 20 mins at room temperature, and fixed cells were washed with PBS. The fixed cells were stained with phenotypic alkaline phosphatase (ALP) staining kit (Chemicon) and alizarin red-S (Sigma) staining solution according to manufacturer’s instruction.

**Figure 2:**
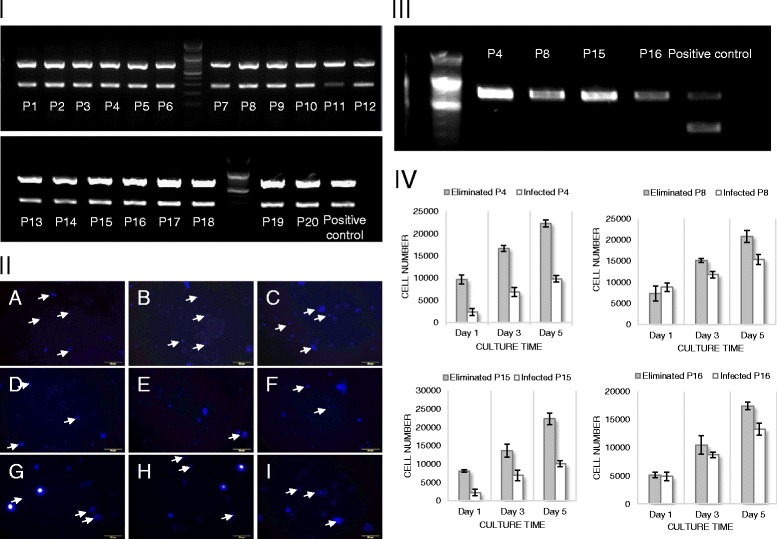
**Mycoplasma-specific PCR analysis and fluorescence staining for primary cultured hSCAPs and proliferation activity.** **I**. PCR analysis for 20 samples. **II**. fluorescence staining of mycoplasma in hSCAPs culture: arrow indicates positive staining of mycoplasma colony. **III**. mycoplasma-specific fluorescence staining of hSCAPs from selected 4 samples before and after mycoplasma elimination process. **IV**. proliferation assay for hSCAPs from selected 4 samples before and after mycoplasma elimination process.

In addition, for 3D osteogenic differentiation, hSCAPs were suspended in 1.1% (w/v) alginic acid (Sigma) and 0.1% (v/v) porcine gelatin (Sigma) solution (all dissolved in PBS, pH 7.4), as described before [19]. Briefly, the cell-gel solution was passed through a peristaltic pump (EYELA) and dropped using a 25-gauge needle into sterile alginate gelation solution composing of 100 mM CaCl_2_ (Sigma), 10 mM HEPES (Sigma), and 0.01% (v/v) Tween (Sigma) at pH 7.4 with stirring. Approximately 10,000 cells were encapsulated in each alginate hydrogel. The hydrogels remained in gently stirred CaCl_2_ solution for 6–10 mins and were then washed with PBS. The hSCAPs containing hydrogels were transferred to in 10 ml vessels of HARV bioreactors (Synthecon) and the vessels was rotated at 25 rpm. Subsequently, 3D osteogenic differentiation was induced using the same osteogenic medium described above. The bioprocess is illustrated in Figure 2 II. After 3D osteogenic differentiation, the sections of hydrogels were stained with alizarin red-S staining solution according to manufacturer’s instruction.

### 2D and 3D neural differentiation of mycoplasma-eliminated hSCAPs

Prior to neural differentiation, primary cultured hSCAPs were expanded in culture medium consisting of DMEM/F12 mixture (1:1, with glucose, L-glutamine, HEPES Buffer, Lonza), 10% FBS, 20 ng/ml epithelial growth factor (EGF, Peprotech), 10 ng/ml basic fibroblast growth factor (FGF2, Peprotech), 1000 unit/ml LIF (Invitrogen) and 1% penicillin/streptomycin. For neural differentiation, hSCAPs was allowed to form neurospheres by plating non-adherent culture dishes in the presence of 100 ng/ml FGF2 in culture medium consisting of DMEM/F12 mixture, 10% FBS, 1000 unit/ml LIF (Invitrogen) and 1% penicillin/streptomycin for 3 days at 37°C in a 5% CO_2_ humidified incubator. The formed neurospheres were replated on laminin (10 μg/ml, Invitrogen)-coated culture dishes and were allowed to direct neural differentiation in neurobasal medium (Gibco) supplemented with 2% B27 (Invitrogen), 1X insulin-transferrin-sodium-selenite (ITS, BD science), 1% penicillin/streptomycin, 30 ng/ml of NGF, 20 ng/ml EGF and 10 ng/ml FGF2 for one week. Subsequently, hSCAPs were maintained in neuroinduction medium consisting of in neurobasal media (Gibco), 1% B27 supplement (Invitrogen), 1% non-essential amino acid (Gibco), 1 X ITS, 100 units/ml penicillin and 100 μg/ml streptomycin, 100 ng/ml GNDF (Peprotech) and 0.5 μM retinoic acid (Sigma). All of the media were replaced twice a week.

In addition, for 3D neural differentiation, hSCAPs which dissociated from neurospheres were mixed with matrigel (BD Bioscience), and the cell/matrigel solution and 1.1% (w/v) alginic acid solution were placed in separated syringes on syringe pump, and the solutions passed through double jet nodule and dropped into sterile alginate gelation solution composing of 100 mM CaCl_2_, 10 mM HEPES, and 0.01% (v/v) Tween at pH 7.4 with mild stirring. The hydrogels remained in gently stirred CaCl_2_ solution for 6–10 mins and were then washed with PBS. The bioprocess is illustrated in Figure 2 IIIE. The hSCAPs/matrigel were placed in inner side and alginate hydrogel were formed to be outer layer enveloping cell/matrigel. The 3D neural differentiation of hSCAPs containing hydrogels was induced by the culture under same culture condition described before. After 2D and 3D neural differentiation, the cells were fixed with 3.7% formaldehyde for 20 mins at room temperature, and neural differentiation was characterized by immunocytochemical staining with primary antibodies such as Ca^2+^/calmodulin-dependent protein kinases II (rabbit anti-human Cam kinase II, Abcam) and βIII-tubulin (mouse anti-human βIII-tubulin, Abcam).

## Result

### Characterization of primary cultured Stem Cells from Apical Papilla (hSCAPs)

Papilla tissues were procured from pre-molar and third molar tooth of patients aged from 6 to 24 which information of the used teeth is noticed in Table 1, and the minced papilla tissues were placed to be adhered on culture flasks. The outgrowth of cells from papilla tissues was detectable in one or two weeks of tissue attachment (Figure 1 IIA). During the expansion of primary isolated cells from apical papilla, two distinguished cell populations were detected under different culture conditions. When the cells were expanded in α-MEM supplemented with 10% FBS, most cells showed fibroblastic morphology, and small population showing spherical shape was found (Figure 1 IIB). Such spherical shaped cell population was largely found during the expansion in the presence of EGF, FGF2 and LIF (Figure 1 IIE). This morphological difference of cell populations showed different molecular expression, and their stem cell characteristic was evaluated by immunocytochemical staining with stem cell markers. As shown in Figure 1 IIC and D, most of fibroblastic cells derived from papilla tissues showed strongly positive reactions against anti-Stro-1and CD44, and, in contrast, spherical shaped cells did not show positive expression of CD44 (Figure 1 IIF), but showed relatively stronger expressions of nestin and CD133 (Figure 1 IIG and H).

**Table 1:** **Information of premolar and third molar teeth used in this study**

**Sample #**	**Sex**	**Age**	**Teeth**	**Sample #**	**Sex**	**Age**	**Teeth**
1	Male	6	Pre-molar	11	Male	19	Third-molar
2	Male	11	Pre-molar	12	Female	23	Third-molar
3	Male	6	Pre-molar	13	Female	24	Third-molar
4	Male	9	Pre-molar	14	Female	12	Pre-molar
5	Male	10	Pre-molar	15	Female	17	Third-molar
6	Male	12	Pre-molar	16	Female	12	Pre-molar
7	Male	11	Pre-molar	17	Female	13	Pre-molar
8	Male	14	Pre-molar	18	Female	11	Pre-molar
9	Male	10	Pre-molar	19	Female	6	Pre-molar
10	Male	14	Pre-molar	20	Female	7	Pre-molar

### Mycoplasma detection and elimination of primary cultured hSCAPs

Mycoplasma contamination of primary cultured hSCAPs was evaluated by PCR analysis and by mycoplasma-specific staining analysis. PCR analysis was done using BioMycoX mycoplasma PCR detection kit which primers is designed to react specifically with the highly conserved coding region in the mycoplasma genome. All expanded hSCAPs which derived from patients aged from 6 to 24 were tested, and, as shown in Figure 2 I, gel running with PCR products showed two bands in all tested samples: one band with approximate size of 700 bp was an internal DNA band to confirm the well PCR reaction, and the other bands which positioned around 250–300 bp indicated mycoplasma contamination. Even though sample number 11 showed relatively week band intensity in comparison with other samples, PCR analysis showed most clear evidence of mycoplasma contamination of all tested hSCAPs.

Accompanied with the analysis of mycoplasma contamination in gene level, mycoplasma contamination was also evaluated by mycoplasma-specific staining analysis using MycoFluor™ mycoplasma detection kit. As shown in Figure 2 II, four samples (patient number 4, 8, 15, and 16) of hSCAPs were selected randomly for staining, and all tested hSCAPs cultures showed positive staining of mycoplasma colony in live cells. Relatively small sized blue fluorescent spots indicate stained mycoplasma colonies within cytosol of hSCAPs, and nucleus within dead cells showed larger fluorescent spots. In addition, mycoplasma-positive staining was frequently detectable around nucleus.

After the detection of mycoplasma contamination via PCR and mycoplasma-specific staining analysis, Mycoplasma was eliminated from the contaminated hSCAPs using BioMycoX® Mycoplasma Elimination Kit. After mycoplasma elimination of randomly selected hSCAPs sample, the presence of mycoplasma was evaluated by mycoplasma-specific staining PCR analysis. As shown in Figure 2 III, the mycoplasma-eliminated hSCAPs culture did not show any bands at approximate size of 250–300 bp in PCR analysis, which indicates well elimination of contaminated mycoplasma.

### Proliferation activity of mycoplasma-contaminated and -eliminated hSCAPs

Four samples (patient number 4, 8, 15, and 16) were selected randomly to evaluate the effect of mycoplasma contamination on cell proliferation, and the proliferation activity of mycoplasma-contaminated and -eliminated hSCAPs was analyzed by BrdU incorporation assay. As shown in Figure 2 IV, although the extent of differences in proliferation activities between mycoplasma-contaminated hSCAPs and mycoplasma-eliminated hSCAPs was varied in the selected samples, mycoplasma-eliminated hSCAPs showed generally much higher proliferation activities than mycoplasma-contaminated hSCAPs throughout whole culture period. Some hSCAPs showed two fold increase in proliferation activity at 5 days of cultures after mycoplasma elimination, which demonstrates the negative effect of mycoplasma contamination on cell proliferation activity.

### Osteogenic and neural differentiation of mycoplasma-eliminated hSCAPs

With mycoplasma-eliminated hSCAPs, 2D and 3D osteogenic and neural differentiation were induced under certain osteogenic and neural differentiation conditions.

**Figure 3:**
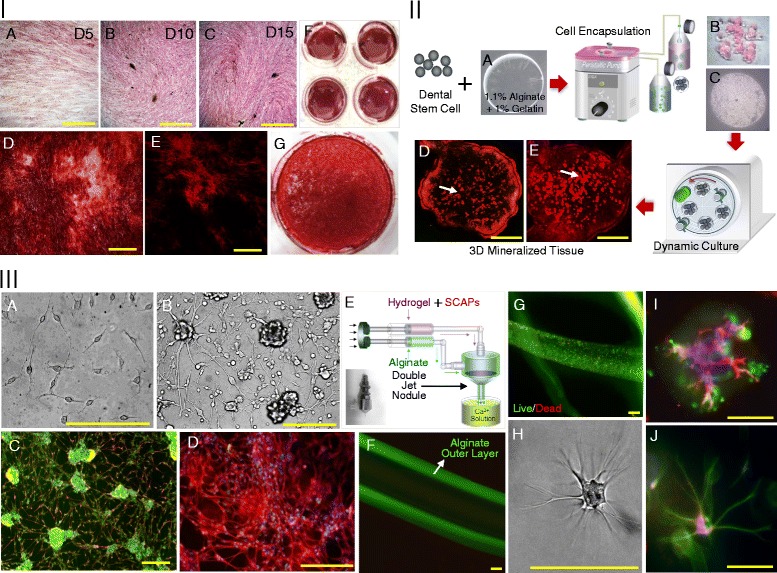
**Osteogenic and neural differentiation of mycoplasma-eliminated hSCAPs. I**. 2D Osteogenic differentiation of mycoplasma-eliminated hSCAPs: **A**, **B** and **C**. Phenotypic ALPase expression after 5, 10 and 15 days of osteogenic culture, **D**, **F** and **G**. Alizarin red-S stained image of mineralized nodules after 20 days of osteogenic culture under light microscope, **E**. alizarin red-S stained image of mineralized nodules after 20 days of osteogenic culture under fluorescence microscope, **II**. 3D Osteogenic differentiation of mycoplasma-eliminated hSCAPs: **A**. alginate hydrogel, **B** and **C**: alginate hydrogel encapsulating hSCAPs, **D** and **E**. alizarin red-S stained image of mineralized nodules within alginate hydrogel after 20 days of 3D osteogenic culture under fluorescence microscope, **III**. 2D and 3D neural differentiation of mycoplasma-eliminated hSCAPs: **A** and **B**. microscopic images of hSCAPs in 2D neural differentiation culture. **C** and **D**. βIII tubulin (red), Cam kinase II (green) and nuclear staining (DAPI; blue), **E**. Schematic illustration of hSCAPs encapsulation of hSCAPs for 3D neural differentiation. **F**. tubular alginate hydrogel, **G**. live and dead image of encapsulated hSCAPs in tubular alginate hydrogel, **H**. microscopic images of hSCAPs in 3D neural differentiation culture, **I**. βIII tubulin (red), Cam kinase II (green) and nuclear staining (DAPI; blue) of differentiated hSCAPs in 3D neural differentiation culture, **J**. βIII tubulin (green), Cam kinase II (red) and nuclear staining (DAPI; blue) of differentiated hSCAPs in 3D neural differentiation culture, Scale bars indicate 200 μm.

For 2D osteogenic differentiation of mycoplasma-eliminated hSCAPs, ostegenic differentiation of hSCAPs was induced in the presence of osteogenic supplements such as ascorbic acid, dexamethasone and β-glycerophosphate, and, as shown in Figure 3 IA-C, culture time-dependent increase of phenotypic ALP expression during osteogenic differentiation. After 20 days of osteogenic differentiation, mineralized nodules were formed in osteogenic culture of hSCAPs throughout whole culture area, which proved by strong positive staining with alizarin red-S (Figure 3 ID, F and G), and such mineralized nodules were found to have high content of calcium and phosphate, which proved by high fluorescence in alizarin red-S staining under fluorescent microscope (Figure 3 IE). In addition to 2D osteogenic differentiation of mycoplasma-eliminated hSCAPs, 3D osteogenic differentiation of hSCAPs were induced by dynamic osteogenic culture of alginate encapsulated hSCAPs in a rotating HARV bioreactor (Figure 3 II). 3D highly mineralized nodules formed by differentiated hSCAPs within alginate hydrogel were found at 20 days of dynamic osteogenic culture (Figure 3 IID and E)

For 2D neural differentiation of mycoplasma-eliminated hSCAPs, hSCAPs were allowed to form neurospheres in the presence of FGF2 and FBS and then hSCAPs-derived neurospheres were plated on laminin-coated culture, 2D neural differentiation was induced by the neural inductive culture in the presence of NGF, EGF, and FGF2 for one week and by the subsequent neural maturation culture in the presence of GDNF and retinoic acid. As shown in Figure 3 IIIA and B, morphological change of hSCAPs to biopolar and multipolar cells was found, and extension of neurite from differentiated hSCAPs was also detected around the plated neurospheres. In addition, the differentiated hSCAPs expressed βIII-tubulin along with the extended neurite and cell bodies and expressed cam kinase II, indicating neural differentiation (Figure 3 IIIC and D). In addition to 2D neural differentiation of mycoplasma-eliminated hSCAPs, neurosphere-derived single cell suspension was mixed with matrigel and encapsulated alginate hydrogel using double jet nodule (Figure 3 IIIE), and 3D neural differentiation was induced under the same culture condition. As shown in Figure 3 IIIF and G, alginate hydrogel well formed the outer layer encapsulating cells, and encapsulated hSCAPs showed well viability within hydrogel, and multipolar cells expressing neural markers such as βIII-tubulin and cam kinase II were found within hydrogel (Figure 3 IIIH-J).

## Discussion

Recently, postnatal stem cells from dental tissues have been received great attention in the field of tissue engineering and regenerative medicine in terms of relatively easy accessibility, multi-differentiation capacity and possible autologus implantation. Implantation studies showed *in vivo* bone and neural tissue regeneration capacity of implanted dental tissue-derived stem cell [13,14,20]. Regarding developmental origin, so called neural crest derived ectomesenchyme, of stem cells to participate in tooth morphogenesis, different lineage differentiation, mesenchymal differentiation and neuroectodermal differentiation, might be derived from postnatal stem cells from dental tissues under certain circumstance [1,2]. The residence of postnatal stem cells showing multi-differentiation capacity have been identified within various dental tissues such as dental pulp, periodontal ligament, and papilla tissues [3,6,7], and, among these dental tissues, papilla tissue which generally found in a developing tooth is known to be a pool of stem cells [21]. In our study, different stem cell populations could be generated by the culture under different culture conditions during expansion of stem cells from apical papilla (hSCAPs), and a stem cell population showing fibroblastic spindle shape during the expansion expressed mesenchymal markers such as Stro-1 and CD44 which consistent to the previous report to show the derivation of stem cells with mesenchymal characteristic from dental papilla tissue [3], but, during hSCAPs expansion in the presence of EGF, FGF2 and LIF, distinct cell population with spherical shape appeared and expressed of neural stem cell markers such as nestin and CD133 [22]. Both expressions of mesenchymal and neural stem cell maker of papilla-derived stem cells might be related to their stem cell characteristics which derived from neural crest-derived ectomesenchyme in tooth developmental process [9,23]. However, it might be necessary to investigate more about the characteristics of postnatal stem cells from dental tissues to broaden their therapeutic applications.

In spite of potent usefulness of stem cells from dental tissues in tissue engineering and regenerative medicine, for clinical application and stem cell researches using postnatal stem cells from dental tissues, bacterial contamination should be considered in fact most teeth are usually extracted due to oral disease such as oral cavity which might be frequently related with bacterial infections. Especially, mycoplasma is one of most popular microorganism frequently found in oral cavity [16], and is the smallest prokaryote with below 1 μm in a diameter which small size allows mycoplasma to pass through conventional filters used to prohibit bacterial and fungal contamination, and mycoplasma easily absorb into host animal cells [15]. In that dental tissues can be frequently exposed to bacterial infection, mycoplasma infection of propagated dental tissue-derived cell cultures is probably unavoidable. In our study, most premolar and third molar teeth were extracted from patient for orthopedic surgery to cure oral disease, and papilla tissues was procured from the discarded teeth. After isolation, mycoplasma contamination was evaluated with primary cultured hSCAPs, and most hSCAPs were found to be contaminated by mycoplasma, which were proved by positive staining and mycoplasma-specific DNA expression. To confirm the possible cross-contamination from other cells in our laboratory, adipose-derived stem cell, human dermal fibroblasts and primary cultured periodontal ligament cells was used to test mycoplasma contamination, but there was no sign of mycoplasma contamination (data not shown). Such mycoplasma contamination is known to affect many aspects of cell physiology such as cell proliferation, chromosomal aberration and also immunological reactions [17,18]. For example, the 50% of inhibition in cell proliferation was reported by influencing nutrient consumption of cells during cell culture [17]. In addition, upon *in vivo* implantation of mycoplasma-contaminated cells, mycoplasma from infected cells might affect host cells participating in immunological reactions such as macrophage activation and inhibition of antigen presentation [18]. In our study, the contaminated mycoplasma in hSCAPs were eliminated, and it was found that mycoplasma contamination affected cell proliferation activity of primary culture hSCAPs. Finally, the mycoplasma-eliminated hSCAPs showed multi-differentiation capacity into osteogenic and neural lineage cells under certain culture conditions, and also 3D osteogenic and neural differentiation could be successfully induced using hydrogel encapsulation technique or dynamic culture system for their application to tissue engineering. Our study might suggest that the experimental process of mycoplasma elimination may be required for primary cell culture and for the application of primary cells to tissue engineering and regenerative medicine, but its time-consuming and expensive process, which takes approximately for 7–10 days, is still a problem to be overcome for its practical application to conventional culture process.

## Conclusion

Our study showed that dental papilla-derived stem cells could be isolated from the discard human premolar and third molar teeth after orthodontic therapy and that almost dental papilla-derived stem cell was shown to be infected by mycoplasma, which mycoplasma infection affected cell proliferation activity. Such infected mycoplasma in dental papilla-derived stem cells could be clearly eliminated using the mycoplasma elimination kit, and mycoplasma-eliminated stem cells showed multi-differentiation activity into osteogenic and neural lineage differentiation. Based on scientific knowledge that infected mycoplasma can affect cell proliferation activity, chromosomal aberration and immune response in cell culture and implantation, mycoplasma detection and elimination process should be considered as one of essential steps prior to stem cell research and potent clinical application using postnatal stem cells from dental tissues.

## Abbreviations

SCAPs: Stem cells from apical papilla
PBS: Phosphate buffered saline
FBS: Fibroblast growth factor
P/S: Penicillin/Streptomycin
DAPI: 4′,6-diamidino-2-phenylindole
PCR: Polymerase chain reaction
ALP: Alkaline phosphatase
EGF: Epithelial growth factor
FGF: Fibroblast growth factor
LIF: Leukemia inhibitory factor
GDNF: Glial cell-derived neurotrophic factor
HARV: High aspect ratio vessel
NGF: Nerve growth factor
